# Molecular Identification and Phylogenetic Position of *Chaunocephalus ferox* (Digenea: Echinostomatidae) from the Asian Openbill (*Anastomus oscitans*) in Thailand

**DOI:** 10.3390/ani16142215

**Published:** 2026-07-16

**Authors:** Chavanut Jaroenchaiwattanachote, Weerachai Saijuntha, Warayutt Pilap, Wangworn Sankamethawee, Chairat Tantrawatpan, Tongjit Thanchomnang

**Affiliations:** 1 Biomedical Science Research Unit, Mahasarakham University, Maha Sarakham 44000, Thailand; chavanut.j@msu.ac.th (C.J.); weerachai.s@msu.ac.th (W.S.); 2 Center of Excellence in Biodiversity Research, Mahasarakham University, Maha Sarakham 44150, Thailand; warayutt@msu.ac.th; 3 Faculty of Medicine, Mahasarakham University, Maha Sarakham 44000, Thailand; 4 Walai Rukhavej Botanical Research Institute, Mahasarakham University, Maha Sarakham 44150, Thailand; 5 Department of Environmental Science, Faculty of Science, Khon Kaen University, Khon Kaen 40002, Thailand; wangsa@kku.ac.th; 6 Division of Cell Biology, Department of Preclinical Sciences, Faculty of Medicine, and Center of Excellence in Stem Cell Research and Innovation, Thammasat University, Rangsit Campus, Pathum Thani 12120, Thailand

**Keywords:** host–parasite interaction, Avian trematodes, wildlife disease surveillance, molecular epidemiology, One Health

## Abstract

*Chaunocephalus ferox* (Rudolphi, 1795) is an intestinal trematode of the family Echinostomatidae that commonly infects waterbirds, including the Asian Openbill (*Anastomus oscitans*). Despite its wide geographic distribution and ecological relevance in migratory birds, molecular data for this species remain limited. This study aimed to characterize the genetic variation and phylogenetic position of *C. ferox* recovered from a deceased Asian Openbill in Thailand. Adult flukes were examined using nuclear (28S rDNA and 5.8S–ITS2) and mitochondrial (cytochrome c oxidase subunit 1; *CO1*) markers. Sequence analyses revealed low intraspecific variation among Thai isolates, with a single variable site detected in each marker. However, comparisons with sequences from Egypt, China, and Ukraine demonstrated measurable genetic divergence, suggesting possible geographic and host-associated structuring among *C. ferox* populations. Phylogenetic analyses based on nuclear and mitochondrial datasets consistently supported the monophyly of *C. ferox* within Echinostomatidae and confirmed its distinct genetic lineage among echinostomes. These findings provide baseline molecular data for a trematode parasite of migratory waterbirds, highlighting the potential role of avian hosts in the long-distance dispersal of helminths across wetland ecosystems. Broader geographic sampling is needed to better understand the population structure and evolutionary dynamics of this parasite.

## Simple Summary

This study examines the molecular characteristics and phylogenetic position of the intestinal trematode *Chaunocephalus ferox*, which infects the Asian Openbill, a widespread migratory waterbird in Southeast Asia. Using nuclear (28S rDNA and ITS2) and mitochondrial (*CO1*) DNA markers, we confirmed species identity and evaluated genetic variation among Thai isolates, as well as comparisons with sequences from other geographic regions. Although genetic variation within Thailand was low, detectable divergence among international isolates suggests geographic structuring of parasite populations. Because Asian Openbills forage in wetlands and agricultural areas, they may contribute to the maintenance and dispersal of trematodes across ecosystems. This study provides baseline molecular data that support wildlife parasite surveillance and contribute to a One Health understanding of parasite transmission at the wildlife–environment interface.

## 1. Introduction

Parasites are integral components of natural ecosystems and play fundamental roles in regulating host populations, structuring communities, and shaping evolutionary processes [1]. In wildlife, parasitic infections can significantly impact host fitness, survival, and reproductive success, ultimately affecting biodiversity and ecosystem stability. Within a One Health framework, parasites of wild animals are increasingly recognized as key indicators of ecosystem health and as potential drivers of disease emergence at the wildlife–environment–human interface.

Avian trematodes are of particular ecological importance because their complex life cycles link aquatic and terrestrial environments through interactions among multiple hosts [2]. Migratory waterbirds can facilitate the long-distance dispersal of trematodes across wetlands, agricultural landscapes, and international flyways, shaping parasite transmission dynamics and population structure at regional and continental scales [3,4].

The genus *Chaunocephalus* Dietz, 1910 comprises intestinal trematodes infecting piscivorous and molluscivorous birds, particularly storks and other wading birds [5,6,7]. *Chaunocephalus ferox* (Rudolphi, 1795) is a digenean trematode with a complex life cycle involving freshwater snails as the first intermediate host and fish or amphibians as the second intermediate host, in which metacercariae develop [7]. Birds, primarily storks, act as definitive hosts and become infected through the ingestion of infected second intermediate hosts. Heavy infections of *C. ferox* are associated with the formation of intestinal nodules, cachexia, and, in severe cases, mortality, highlighting its importance for avian health and conservation [8,9].

The Asian Openbill (*Anastomus oscitans*) is one of the most abundant wetland birds in mainland Southeast Asia and has undergone rapid population expansion and range shifts over recent decades [10,11,12]. This species inhabits rice paddies, floodplains, and wetlands and feeds predominantly on freshwater snails, especially the invasive golden apple snail *Pomacea canaliculata* [13]. Through this specialized feeding behavior, Asian Openbills play an important ecological role in regulating snail populations, including species that serve as intermediate hosts for trematodes, but are simultaneously exposed to infection, positioning them as key definitive hosts within freshwater parasite transmission cycles [14].

Previous studies have documented diverse trematode assemblages in *A. oscitans*, including species of *Echinostoma*, *Echinochasmus*, *Clinostomum*, *Allechinostomum*, *Cathaemasia*, and *Chaunocephalus* [5,15,16,17]. However, despite repeated reports of *C. ferox* infections in storks across different regions, molecular data for this species remain limited, particularly from Asian populations. Moreover, the population genetic structure of *C. ferox* and the extent to which migratory birds influence its dispersal remain poorly understood.

Molecular tools using nuclear and mitochondrial DNA markers have become essential for parasite identification, genetic characterization, and phylogenetic inference [18,19]. Mitochondrial markers such as cytochrome c oxidase subunit 1 (*CO1*) are widely used to detect genetic variation and geographic structuring [20], whereas nuclear ribosomal regions, including 28S rDNA and internal transcribed spacers (ITS), provide complementary resolution for species delimitation and evolutionary relationships [21,22]. Integrating these markers is particularly valuable for wildlife disease surveillance and for understanding parasite transmission dynamics under a One Health framework.

Therefore, the present study aimed to genetically characterize *C. ferox* infecting Asian Openbills in Thailand using nuclear (28S rDNA and ITS2) and mitochondrial (*CO1*) DNA markers and to evaluate its phylogenetic position within the family Echinostomatidae. By providing new molecular data for a trematode parasite of migratory birds, this study contributes to wildlife parasite surveillance and enhances understanding of parasite transmission at the wildlife–environment interface in Southeast Asia.

## 2. Materials and Methods

### 2.1. Sample Collection

**Figure 1:**
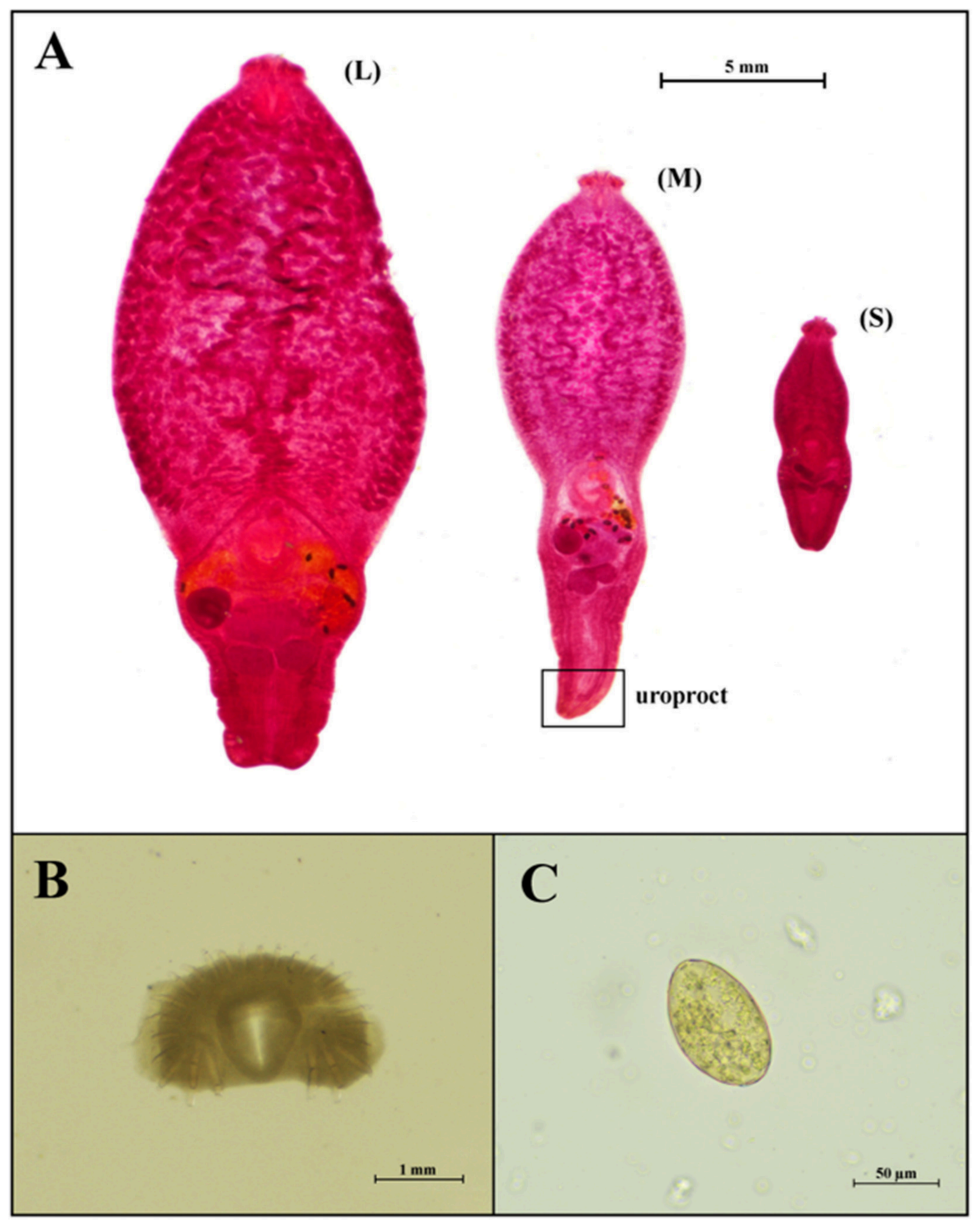
Adult *Chaunocephalus ferox* recovered from the intestine of an Asian Openbill and stained with carmine. (**A**) Three body-size classes of *C. ferox* were observed: large (L), medium (M), and small (S). The boxed area indicates the uroproct. (**B**) Anterior view of the head collar showing 27 collar spines. (**C**) Egg.

The specimens of *C. ferox* were collected from the intestine of a deceased Asian Openbill provided by a farmer in Kerng Subdistrict, Maung District, Maha Sarakham Province, Thailand. The intestine was removed, opened longitudinally with scissors, and examined for adult flukes. A heavy infection of *C. ferox* was observed. Based on body size, the recovered adult flukes were classified into three size categories, namely large (L), medium (M), and small (S) (Figure 1). Adult flukes were collected and washed several times in sterile normal saline until all visible debris had been removed. The specimens were then preserved in 70% ethanol and stored at −20 °C until DNA extraction for subsequent molecular analyses. After washing, several specimens from each size class were randomly selected for carmine staining (Figure 1). Morphological identification was performed according to the diagnostic criteria described by Choe et al. [5], Greben et al. [17], and Mehra [23].

### 2.2. Molecular Analysis

Five worms of each size, totaling 15 specimens, were randomly selected for molecular analysis. The worms were washed in sterile normal saline several times before being kept in 80% alcohol until further required. DNA was extracted from each adult worm by using E.Z.N.A.^®^ Tissue DNA kit (Omega Bio-tek, Norcross, GA, USA) following the manufacturer’s protocol. The *CO1* and ITS2 regions were amplified by using the primers, PCR condition, and protocol published by Tantrawatpan et al. [24], while the 28S rDNA region followed the protocol published by Tkach et al. [21].

### 2.3. Data Analysis

The sequences were checked and edited using ABI sequence scanner version 1.0 and BioEdit version 7.2.6 [25]. Multiple sequence alignments were performed using MAFFT version 7 [26]. Phylogenetic relationships were carried out using Maximum Likelihood (ML) and Bayesian Inference (BI) approaches. The most appropriate nucleotide substitution models were determined with MrModeltest version 2 [27] based on the Akaike Information Criterion (AIC), identifying GTR+I+G for *CO1* and 28S rDNA and GTR+G for the 5.8S–ITS2 region. Maximum Likelihood analyses were conducted in raxmlGUI version 2.0 [28,29], which serves as a graphical interface for RAxML, using 1000 rapid bootstrap replicates under the selected models. Bayesian analyses were performed in MrBayes version 3.2.7 [30], with parameter settings configured in Mesquite version 3.81 [31]. Two independent Markov chain Monte Carlo (MCMC) runs were executed, each comprising four chains, for ten million generations, with trees sampled every 1000th generations. The analyses were continued until the average standard deviation of split frequencies fell below 0.01. Convergence diagnostics were evaluated by examining effective sample size (ESS) values in Tracer version 1.7.2 [32]. After discarding the first 25% of sampled trees as burn-in, Bayesian posterior probabilities (BPPs) were calculated, and a 50% majority-rule consensus tree was constructed and visualized using the Interactive Tree of Life version 7.5.1 (iTOL) on a web server (https://itol.embl.de/, accessed on 18 April 2026) [33]. The final phylogram presents ML bootstrap support values (≥50%) alongside BPP values (≥0.50) at the corresponding nodes. To investigate interspecific divergence, pairwise distances for each genetic marker were independently calculated in MEGA X using uncorrected pairwise distances (p-distance) and the Kimura 2-parameter (K2P) model [34].

## 3. Results

### 3.1. Morphological Characteristics of Chaunocephalus ferox

Based on body size, the specimens were classified into three size morphotypes: large (L), medium (M), and small (S). The L morphotype measured 4.158–5.564 mm (mean 4.913 mm) in body length, with forebody and hindbody widths of 2.237–2.606 mm (mean 2.451 mm) and 1.326–1.475 mm (mean 1.420 mm), respectively. The M morphotype measured 3.473–4.308 mm (mean 3.990 mm) in length, with forebody and hindbody widths of 1.420–1.550 mm (mean 1.490 mm) and 0.865–0.899 mm (mean 0.882 mm), respectively. The S morphotype ranged from 1.825 to 2.683 mm (mean 2.243 mm) in length, with forebody and hindbody widths of 0.368–1.072 mm (mean 0.677 mm) and 0.551–0.742 mm (mean 0.612 mm), respectively (Figure 1A). All specimens possessed a prominent cephalic collar bearing 27 collar spines surrounding the oral sucker (Figure 1B). Eggs were oval, measuring 72–81 × 50–56 μm (mean 76 × 52 μm) (Figure 1C). The intestinal caeca united with the excretory vesicle to form a uroproct with a terminal excretory opening.

Morphologically, the Thai specimens closely matched the descriptions of *Chaunocephalus ferox* from India [23] and Korea [5], particularly in the arrangement of the testes, ovary, Mehlis’ gland, and the presence of a uroproct. However, the Thai specimens were considerably smaller than those reported from Korea (8.030–8.091 mm in body length) and showed slight differences in testicular size. The Mehlis’ gland was located between the ovary and testes, although its margins were indistinct because of intense staining. Vitelline follicles extended laterally from the posterior margin of the pharynx to the level of the testes but did not reach the posterior extremity (Table S1). Compared with *C. similiferox*, most morphological structures of the Thai specimens were smaller, except for the hindbody width and testes, which were slightly larger (Table S1).

### 3.2. DNA Sequence Analysis

All nuclear (28S rDNA and 5.8S–ITS2) and mitochondrial (*CO1*) sequences generated in this study were successfully obtained from 15 adult *C. ferox* specimens representing three body-size classes (large, medium, and small). The sequences were deposited in GenBank under accession numbers PV270125–PV270139 (28S rDNA), PV270161–PV270173 (ITS2), and PV270095–PV270108 (*CO1*).

#### 3.2.1. Mitochondrial CO1 Sequence

**Scheme 1:**
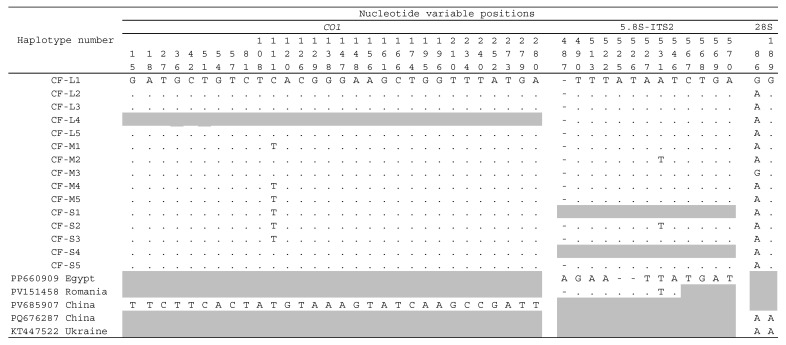
Nucleotide variable positions of mitochondrial *CO1*, nuclear 5.8S–ITS2, and 28S rDNA sequences among *Chaunocephalus ferox* specimens from Thailand and reference isolates from GenBank. Dots (.) indicate nucleotide identity with the reference sequence (CF-L1), whereas dashes (–) represent alignment gaps (insertion/deletion positions). Gray shaded areas indicate regions not included or unavailable in the alignment. Sample codes beginning with CF represent *C. ferox* specimens from Thailand, whereas PP660909, PV151458, PV685907, PQ676287, and KT447522 are reference sequences retrieved from GenBank.

The aligned *CO1* dataset comprised a 280 bp fragment from 14 *C. ferox* sequences from Thailand and one sequence from China (GenBank accession no. PV685907). A total of 30 polymorphic sites were identified across all 15 sequences, including 10 purine transitions, 11 pyrimidine transitions, and 9 transversions (Scheme 1), resulting in an overall nucleotide divergence of 10.71%. In contrast, analyses restricted to the Thai specimens revealed only a single pyrimidine transition at position 111, corresponding to a low nucleotide divergence of 0.36% across all size classes.

#### 3.2.2. Nuclear Ribosomal DNA Sequence

The 28S rDNA alignment included 549 bp from Thai *C. ferox* specimens and two reference sequences from China (PQ676287) and Ukraine (KT447522) in infected oriental and black storks (*Ciconia nigra*), respectively. Two purine transition substitutions were detected at positions 86 and 189, corresponding to a divergence of 0.36%. Notably, all Thai sequences differed consistently from both Chinese and Ukrainian sequences at position 189, whereas no variation was observed among the Thai specimens themselves.

The 5.8S–ITS2 alignment comprised 582 bp and included 13 sequences generated in this study, along with two reference sequences retrieved from GenBank from Egypt (PP660909) and Romania (PV151458), obtained from a wild bird and a white stork (*Ciconia ciconia*), respectively. A total of 13 variable sites (2.23%) were detected among the Thai, Egyptian, and Romanian isolates. These variations consisted of one pyrimidine transition at position 567, one purine transition at 569, eight transversion substitutions at positions 490, 513, 522, 527, 531, 546, 568, and 570, and three insertion–deletion events (indels). The indels included one insertion at position 487 and three deletions at positions 525 and 526 in the Egyptian sequence. Low intraspecific variation was observed among the Thai ITS2 sequences, with a single transversion detected at position 531 (0.17%).

### 3.3. Genetic Differences and Phylogenetic Tree Analyses

Genetic divergence analyses based on the nuclear markers 28S rDNA and 5.8S–ITS2 revealed clear patterns of intra- and intergeneric variation within the family Echinostomatidae. Mean intergeneric pairwise distances ranged from 2.56% to 6.51% for 28S rDNA and from 3.04% to 17.55% for 5.8S–ITS2. In contrast, the mitochondrial *CO1* marker exhibited substantially higher genetic divergence, with pairwise distances ranging from 20.33% to 28.04% relative to other available echinostomatid taxa. Intraspecific genetic divergence among 28S rDNA sequences of *C. ferox* from Thailand (this study), China (P676287), and Ukraine (KT447522) was very low, ranging from 0.00% to 0.21% under both p-distance and K2P models (Table S2). The 5.8S–ITS2 dataset demonstrated slightly higher intraspecific variation, with divergence values ranging from 0.19% to 1.14%, including sequences from Egypt (PP660909) and Romania (PV151458), suggesting some degree of geographic differentiation while maintaining species-level cohesion. Moreover, this marker showed greater interspecific divergence within Echinostomatidae, ranging from 3.04% to 17.55% and 3.10% to 20.21% for p-distance and K2P, respectively (Table S3).

**Figure 2:**
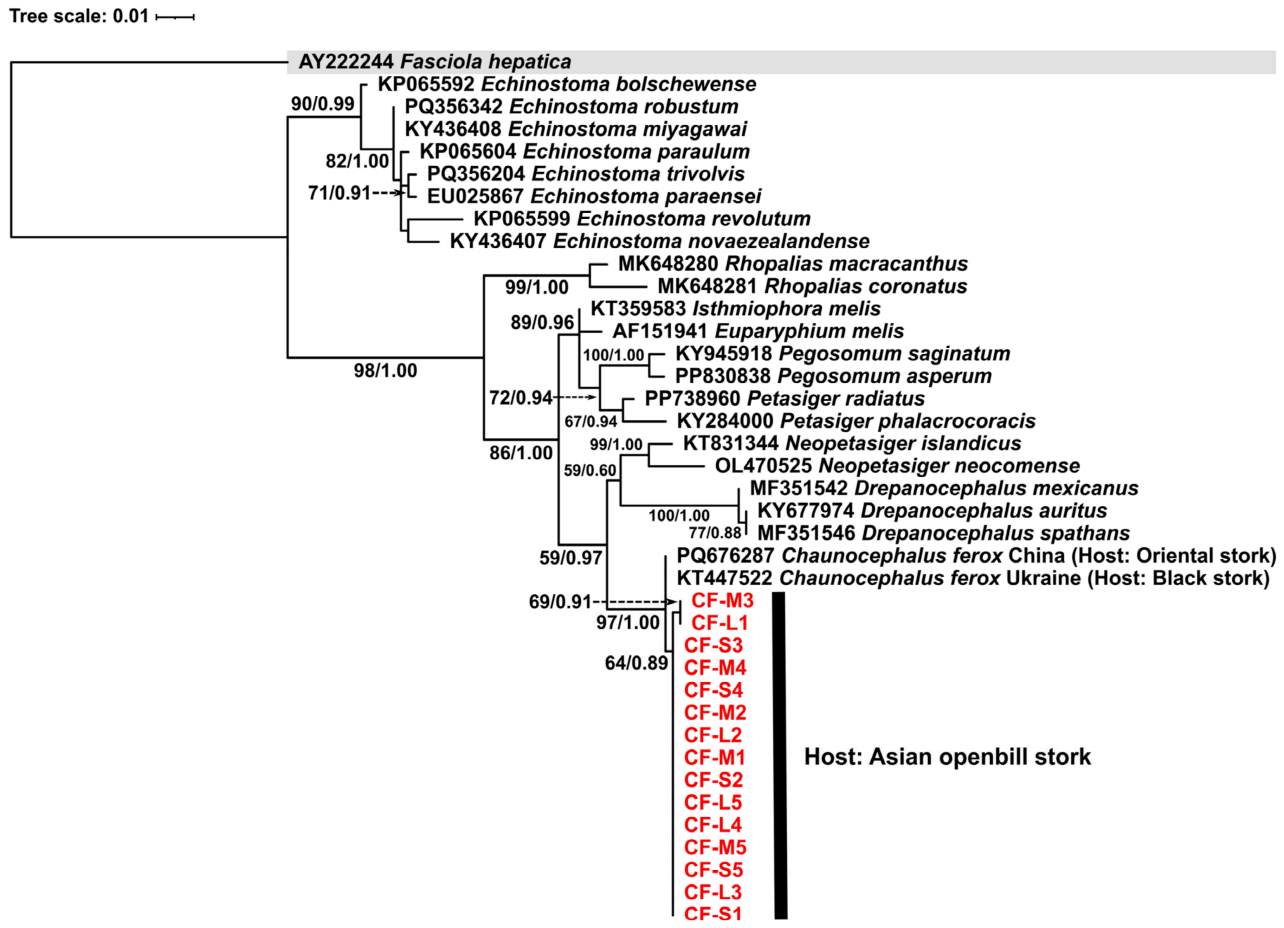
Maximum likelihood tree constructed based on 28S rDNA sequences. Nodal supports are the bootstrap values generated by maximum likelihood and Bayesian inference analyses. The taxon labeled CF in red represents *Chaunocephalus ferox*, examined in this study. The gray shaded area highlights the outgroup.

**Figure 3:**
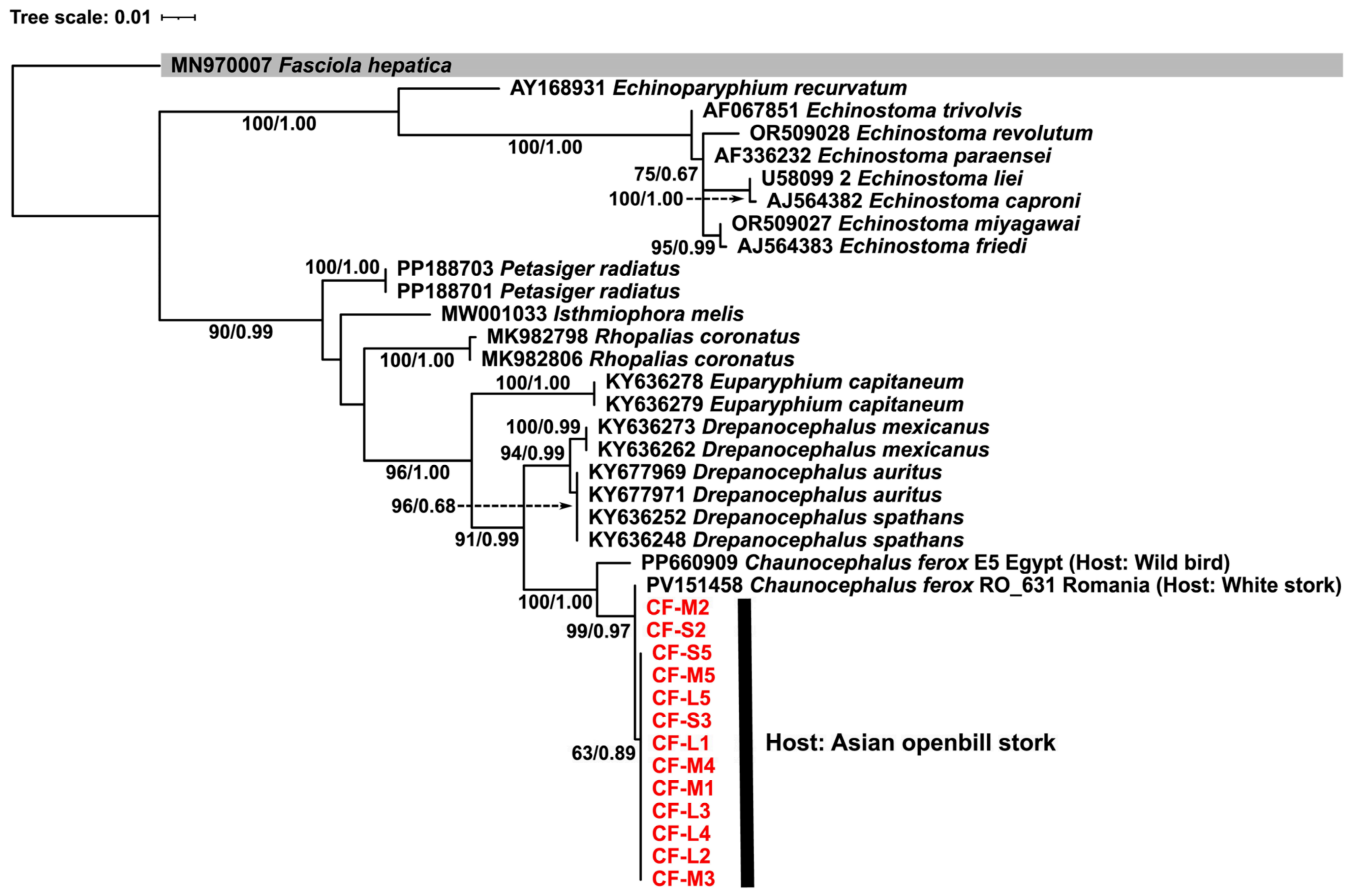
Maximum likelihood tree constructed based on 5.8S–ITS2 sequences. Nodal supports are the bootstrap values generated by maximum likelihood and Bayesian inference analyses. The taxon labeled CF in red represents *Chaunocephalus ferox*, examined in this study. The gray shaded area highlights the outgroup.

**Figure 4:**
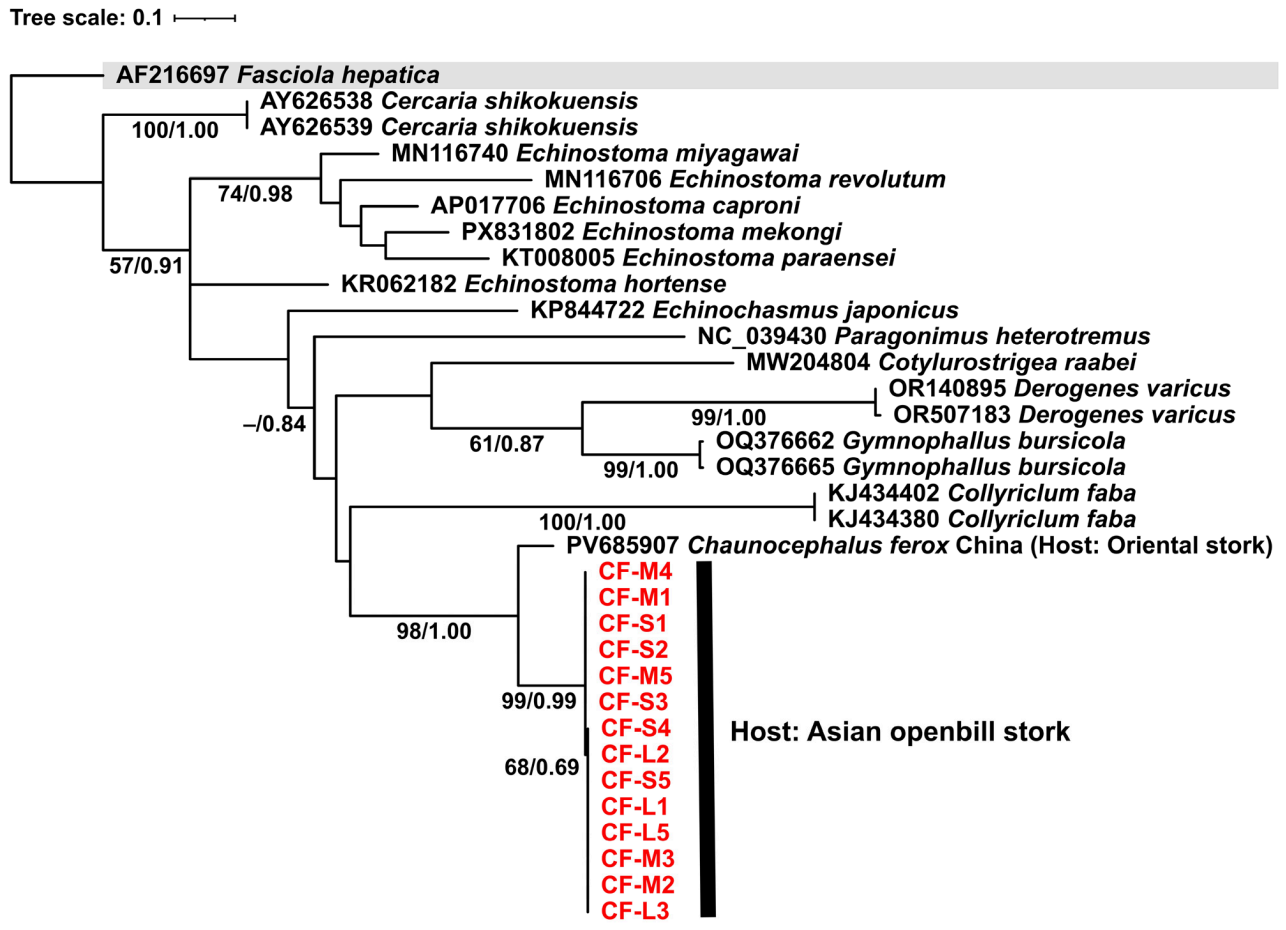
Maximum likelihood tree constructed based on *CO1* sequences. Nodal supports are the bootstrap values generated by maximum likelihood and Bayesian inference analyses. The taxon labeled CF in red represents *Chaunocephalus ferox*, examined in this study. The gray shaded area highlights the outgroup.

Maximum likelihood and Bayesian inference phylogenetic analyses based on 28S rDNA, 5.8S–ITS2, and *CO1* sequences consistently supported the monophyly of *C. ferox* within the family Echinostomatidae (Figure 2, Figure 3 and Figure 4). In both the 28S rDNA and ITS2 trees, all *C. ferox* sequences formed a well-supported clade clearly distinct from other echinostomatid genera. Phylogenetic analyses based on nuclear markers positioned *C. ferox* in close relationship with the genera *Petasiger*, *Neopetasiger*, *Isthmiophora*, *Euparyphium*, *Rhopalias*, *Pegosomum*, *Drepanocephalus*, and *Echinostoma*, forming a distinct lineage within the family. Additional *C. ferox* sequences from different geographic regions clustered within the same clade, further supporting species-level cohesion. Other echinostomatid genera, including *Drepanocephalus*, *Isthmiophora*, *Petasiger*, *Rhopalias*, *Euparyphium*, and *Echinostoma*, also formed distinct and well-supported clades across both nuclear-based phylogenies. The trees were rooted using *Fasciola hepatica*, and bootstrap and posterior probability values were generally high, indicating strong support for the inferred phylogenetic relationships.

The *CO1*-based phylogeny likewise confirmed the distinct genetic lineage of *C. ferox* within the Echinostomatidae. Although all *C. ferox* sequences were clearly separated from other *Echinostoma* species, the Thai isolates formed a separate clade from the Chinese *C. ferox* (PV685907), with strong bootstrap and Bayesian posterior probability (BPP) support and relatively high genetic divergence (10.56–11.65% for p-distance and K2P, respectively). Interestingly, Thai *C. ferox* exhibited a similar level of genetic divergence from the echinostomatid taxon *Echinochasmus japonicus* (KP844722) as from *Collyriclum faba* (Collyriclidae), with an interspecific p-distance of 20.98% (Table S4). This pattern indicates an overlap between intra- and intergeneric genetic divergence. However, the 280 bp partial *CO1* fragment analyzed in this study is relatively short and, together with the limited availability of comparative *CO1* sequences for Echinostomatidae, provides lower phylogenetic resolution than the nuclear markers (28S rDNA and 5.8S–ITS2). Nevertheless, *C. ferox* was consistently recovered as a distinct, well-supported clade in the *CO1* phylogenetic analysis (Figure 4).

## 4. Discussion

*Chaunocephalus* species are distinguished by a bulbous forebody, a short cylindrical hindbody, a long esophagus, and a collar bearing 25–29 spines [23]. The Thai specimens exhibited the diagnostic features of the genus, including 27 collar spines (four angle spines on each side and 19 dorsal and lateral spines), as well as the characteristic arrangement of the reproductive organs and the presence of a uroproct, all of which agree with previous descriptions of *C. ferox* [5,23]. Although *C. similiferox* has been regarded either as a synonym of *C. ferox* [23,35] or as a valid species based on morphological differences [36], the Thai specimens were morphologically more consistent with *C. ferox*. While some measurements differed slightly from those reported for *C. similiferox*, these differences fall within the range of intraspecific variation and do not support species separation. Overall, the morphological evidence confirms the identification of the Thai specimens as *C. ferox*.

Asian Openbills are highly specialized molluscivorous birds and are continuously exposed to trematode infections through the consumption of infected freshwater organisms, particularly snails, fish, and crustaceans [13,14]. Their increasing population size and expanding geographic range across Southeast Asia suggest sustained opportunities for parasite transmission and maintenance within wetland ecosystems [10,11,12]. From a wildlife health perspective, persistent infections with intestinal trematodes such as *C. ferox* may have sublethal effects on host condition and fitness, particularly under conditions of high parasite burden or environmental stress. Severe infections with *C. ferox* have previously been associated with intestinal pathology, cachexia, and mortality in storks, underscoring the importance of monitoring this parasite in wild bird populations [8,9].

The present study provides new molecular data on *C. ferox* infecting the Asian Openbill in Thailand and contributes to a broader understanding of parasite diversity and transmission in wetland-associated wildlife. Comparative analyses of ITS2 and 28S rDNA sequences revealed clear genetic divergence between Thai *C. ferox* and isolates from Egypt, China, and Ukraine deposited in GenBank. The relatively higher divergence observed in the ITS2 region supports its usefulness for detecting geographic structuring and intraspecific variation in echinostomatid trematodes [18,22]. Based on *CO1* sequence comparisons, *C. ferox* from China also exhibited clear genetic differentiation from Thai *C. ferox*. Such geographic genetic differentiation may reflect historical isolation, limited gene flow among parasite populations, or variation in host assemblages and ecological conditions across regions. These findings are consistent with previous studies demonstrating spatial genetic structure in echinostomes and other trematodes with complex life cycles [37,38].

Interestingly, *C. ferox* isolates from different countries were collected from different stork hosts, namely the Asian Openbill (*A. oscitans*) in Thailand and the Oriental Stork (*C. boyciana*) in China, White Stork (*C. ciconia*) from Romania, and Black Stork (*C. nigra*) from Ukraine. Therefore, the observed genetic differentiation may not be explained solely by geographic distance, but may also reflect host-associated structuring. However, broader sampling from the same host species across different geographic regions is required to properly test this hypothesis. Differences in host ecology, feeding behavior, migration patterns, and exposure to distinct intermediate host communities may influence parasite transmission dynamics and contribute to genetic structuring among parasite populations [39,40]. Similar patterns of host-associated genetic differentiation have been reported in other trematodes and parasites with complex life cycles, in which both geographic isolation and host specificity play important roles in shaping population structure [37,38].

Within a One Health framework, migratory birds such as the Asian Openbill represent important biological links connecting aquatic ecosystems, agricultural landscapes, and wildlife habitats. Because parasites are largely constrained by host movement, long-distance avian migration can strongly influence parasite dispersal and population connectivity [3,4]. The detection of genetically distinct *C. ferox* lineages across continents suggests that, although birds facilitate parasite spread, environmental barriers, host specificity, and intermediate host availability may limit homogenization among parasite populations. Similar patterns have been reported for other echinostomatid trematodes, including *Echinostoma revolutum*, *E. miyagawai*, and *Artyfechinostomum malayanum*, where genetic structuring reflects ecological and geographic constraints on transmission [38,41].

Phylogenetic analyses based on nuclear (28S rDNA and ITS) and mitochondrial (*CO1*) markers consistently supported the monophyly of *C. ferox* within the family Echinostomatidae. Nuclear markers grouped *C. ferox* with echinostomatids such as *Petasiger* and *Neopetasiger*, whereas mitochondrial *CO1* sequences showed substantial divergence (>20%) from other available echinostomatid sequences. However, this result should be interpreted cautiously because the analyzed *CO1* fragment was relatively short and comparative mitochondrial data for echinostomatids remain limited in public databases. Such incongruence between nuclear and mitochondrial phylogenies has been reported in other trematode groups and may reflect differences in evolutionary rates, lineage sorting, or historical host associations [22]. Nevertheless, the distinct placement of *C. ferox* across all analyses reinforces its taxonomic validity and highlights the value of integrating multiple genetic markers for parasite systematics.

From a wildlife disease surveillance perspective, the limited genetic variation observed among Thai *C. ferox* isolates contrasts with the divergence detected at broader geographic scales, emphasizing the need for expanded sampling across the Asian–Australasian flyway. Generating baseline genetic data for parasites of migratory birds is critical for detecting changes in parasite distribution, identifying emerging transmission hotspots, and assessing the potential impacts of environmental change on host–parasite systems. Wetland modification, agricultural intensification, and the spread of invasive snail species may further alter trematode transmission dynamics, with implications for wildlife health and ecosystem functioning [13,14].

## 5. Conclusions

This study provides new molecular data on *C. ferox* infecting Asian Openbills in Thailand and confirms its phylogenetic placement within the family Echinostomatidae using nuclear and mitochondrial DNA markers. Although genetic variation among Thai isolates was low, comparisons with geographically distant populations revealed evidence of genetic divergence. These findings highlight the importance of integrating molecular approaches into wildlife parasite surveillance and support a One Health perspective linking migratory birds, wetland ecosystems, and parasite transmission dynamics. Expanded geographic sampling across migratory flyways will be essential to better understand the population structure, dispersal patterns, and epidemiological significance of this trematode.

## Data Availability

The sequences generated in this study were deposited in GenBank under accession numbers PV270125–PV270139, PV270161–PV270173, and PV270095–PV270108.

## Supplementary Materials

The following supporting information can be downloaded at: https://www.mdpi.com/article/10.3390/ani16142215/s1, Table S1: Comparative morphometric data for *Chaunocephalus ferox* from the present study and previous studies, including its putative synonym *C. similiferox* Verma, 1936. Table S2: Pairwise distance matrix of 28S rDNA sequences of Chaunocephalus ferox recovered from Asian openbill storks collected in Thailand, Oriental storks from China, Black storks from Ukraine, and other Echinostomatidae species, showing Kimura 2-parameter distances (upper triangle) and uncorrected pairwise distances (lower triangle). Table S3: Pairwise distance matrix of 5.8S-ITS2 sequences of *Chaunocephalus ferox* recovered from Asian openbill storks collected in Thailand, Wild bird from Egypt, White stork from Romania, and other Echinostomatidae species, showing Kimura 2-parameter (upper triangle) and uncorrected pairwise distances (lower triangle) values. Table S4: Pairwise distance matrix of *CO1* sequences of *Chaunocephalus ferox* recovered from Asian openbill storks collected in Thailand, Oriental storks from China, and other Echinostomatidae species, showing Kimura 2-parameter (upper triangle) and uncorrected pairwise distances (lower triangle) values.
